# Rational design and structure-based engineering of alkaline pectate lyase from *Paenibacillus* sp. 0602 to improve thermostability

**DOI:** 10.1186/s12896-021-00693-8

**Published:** 2021-05-03

**Authors:** Zhanping Zhou, Xiao Wang

**Affiliations:** 1 Tianjin Sinonocy Biological Technology Co. Ltd., Tianjin, 300308 China; 2 Nanfang College of Sun Yat-Sen University, Guangzhou, 510970 China

**Keywords:** Protein engineering, Alkaline pectate lyase, Thermostability, PoPMuSiC, Site-directed mutagenesis

## Abstract

**Background:**

Ramie degumming is often carried out at high temperatures; therefore, thermostable alkaline pectate lyase (PL) is beneficial for ramie degumming for industrial applications. Thermostable PLs are usually obtained by exploring new enzymes or reconstructing existing enzyme by rational design. Here, we improved the thermostability of an alkaline pectate lyase (PelN) from *Paenibacillus* sp. 0602 with rational design and structure-based engineering.

**Results:**

From 26 mutants, two mutants of G241A and G241V showed a higher thermostability compared with the wild-type PL. The mutant K93I showed increasing specific activity at 45 °C. Subsequently, we obtained combinational mutations (K93I/G241A) and found that their thermostability and specific activity improved simultaneously. The K93I/G241A mutant showed a half-life time of 15.9 min longer at 60 °C and a melting temperature of 1.6 °C higher than those of the wild PL. The optimum temperature decreased remarkably from 67.5 °C to 60 °C, accompanied by a 57% decrease in *Km* compared with the *Km* value of the wild-type strain. Finally, we found that the intramolecular interaction in PelN was the source in the improvements of molecular properties by comparing the model structures. Rational design of PelN was performed by stabilizing the α-helices with high conservation and increasing the stability of the overall structure of the protein. Two engineering strategies were applied by decreasing the mutation energy calculated by Discovery Studio and predicting the free energy in the process of protein folding by the PoPMuSiC algorithm.

**Conclusions:**

The results demonstrated that the K93I/G241A mutant was more suitable for industrial production than the wild-type enzyme. Furthermore, the two forementioned strategies could be extended to reveal engineering of other kinds of industrial enzymes.

**Supplementary Information:**

The online version contains supplementary material available at 10.1186/s12896-021-00693-8.

## Background

Pectate lyase (Pel, EC 4.2.2.2) is one pectin depolymerase that catalyzes the cleavage of α-1,4 linkages and produces 4,5-unsaturated oligogalacturonate [1–3]. Bio-scouring is one of the most important industrial applications of alkaline Pels [2, 4, 5]. Bio-scouring shows obvious advantages than conventional chemical degumming [6–9]. In textile industry, degumming process is usually executed at the temperature from 40 °C to 70 °C and in alkaline condition of pH (8–11) [10]. Therefore, it is desirable for Pels to keep stable and active under the corresponding thermal and alkaline conditions for industrial application.

An alkaline Pel (*pelN*) from *Paenibacillus* sp. 0602 has been overexpressed and characterized in a previous study [1]. PelN shows excellent characteristics in ramie degumming combined chemical treatment [11, 12]. However, PelN is unstable at optimal temperature of 65 °C and lead to lower efficiency substantially [1]. Therefore, thermostability is a major obstacle on PelN application in industrial degumming.

Many techniques have been developed to improve protein thermostability in view of site-directed mutagenesis and regional substitution [10, 13–16]. Residue mutations from glycine or proline to alanine with high helix propensity can improve thermostability [17–21]. The PoPMuSiC algorithm has become a powerful tool in protein rational design and protein engineering in recent years. It can predict the thermodynamic stability variation induced by single-site mutation with coefficients depending on the solvent accessibility of the alterant residue [22–24].

In this study, the three strategies mentioned above for protein engineering were applied to improve the stability of PelN. A total of 14 residues were selected as engineering targets for potential mutant screening, and additional combinational mutations were generated from site-direct mutations. Then, we measured the thermostability and other biochemical features of the mutants and made comparisons with those of the wild PelN. Finally, we built 3D structural models of all proteins to interpret the mechanisms for changes in biochemical features, especially thermostability improvement. The strategies described here could be practiced in engineering of other industrial enzymes.

## Results

### Identification of target residues for thermostability improvement

**Fig. 1:**
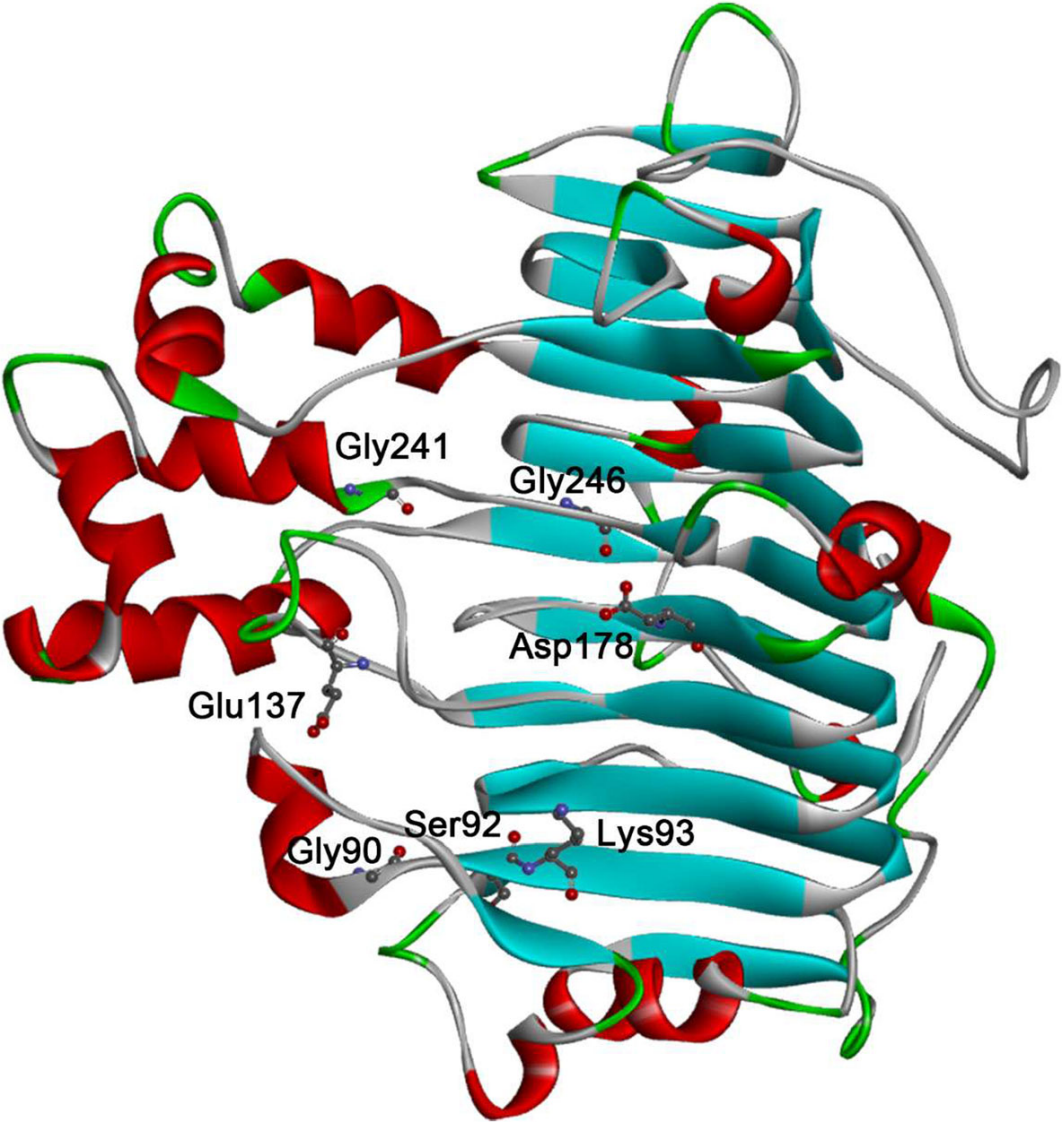
The selected residues for possible stability enhancement by rational design. Discovery studio and the PoPMuSiC algorithm are applied for the mutation energy calculation

It is essential for us to identify the target residues for site-directed mutagenesis to obtain thermostable PelN mutants. First, we found that Gly90, Gly241, Gly316, Pro84, and Pro219 were located in the α-helix (Fig. 1). These residues were reported to destabilize the α-helix structure of proteins [18]. Thus, it benefits stability of α-helix structure [19] to replace Gly90, Gly241, Gly316, Pro84, Pro219 by alanine and valine. To confirm the residues replacement effect on stability, we applied Discovery Studio 4.1 to calculate the mutation energy. The results predicted that Gly90Val, Gly241Ala and Gly241V have a positive effect on stability (see Table S1).

A previous report indicated potential substitutions by the PoPMuSiC algorithm [24]. The ΔΔG of amino acid substitution was obtained by PoPMuSiC analysis (see Table S2). Based on the calculation results, we selected seven residue substitutions of PelN (Glu137Tyr, Glu137Phe, Glu137Trp, Gly246Trp, Gly325Trp, Ser92Phe, and Asp178Phe) to improve the enzyme thermostability. There are also too many substitutions whose ΔΔG values are less than − 1.0 kcal/mol screened by PoPMuSic (see Table S3). Therefore, we used Discovery Studio 4.1 for further screening with the function of Calculate Mutation Energy in this group. The results showed that Lys93Ile, Asp178Phe, Asp178Tyr, Asp178Cys and Asp178Val may have a positive effect on the thermostability of PelN.

### Thermostability of beneficial mutants

Based on the above analysis, 14 potential mutations for stability enhancement were constructed through overlap extension PCR [25]. All PelN variants were expressed in *E. coli* BL21 (DE3) after verification of the gene sequences. The expression of all variants was similar to that of the wild PelN (Fig. S1). Then, we sonicated the collected cells, removed the cell debris by centrifugation and assayed the enzymatic activity of the supernatants. The enzyme activity of the D178C, D178F, D178Y and G246W mutants decreased badly (Fig. S2). The other variants were further screened for positive mutations at 60 °C.

**Fig. 2:**
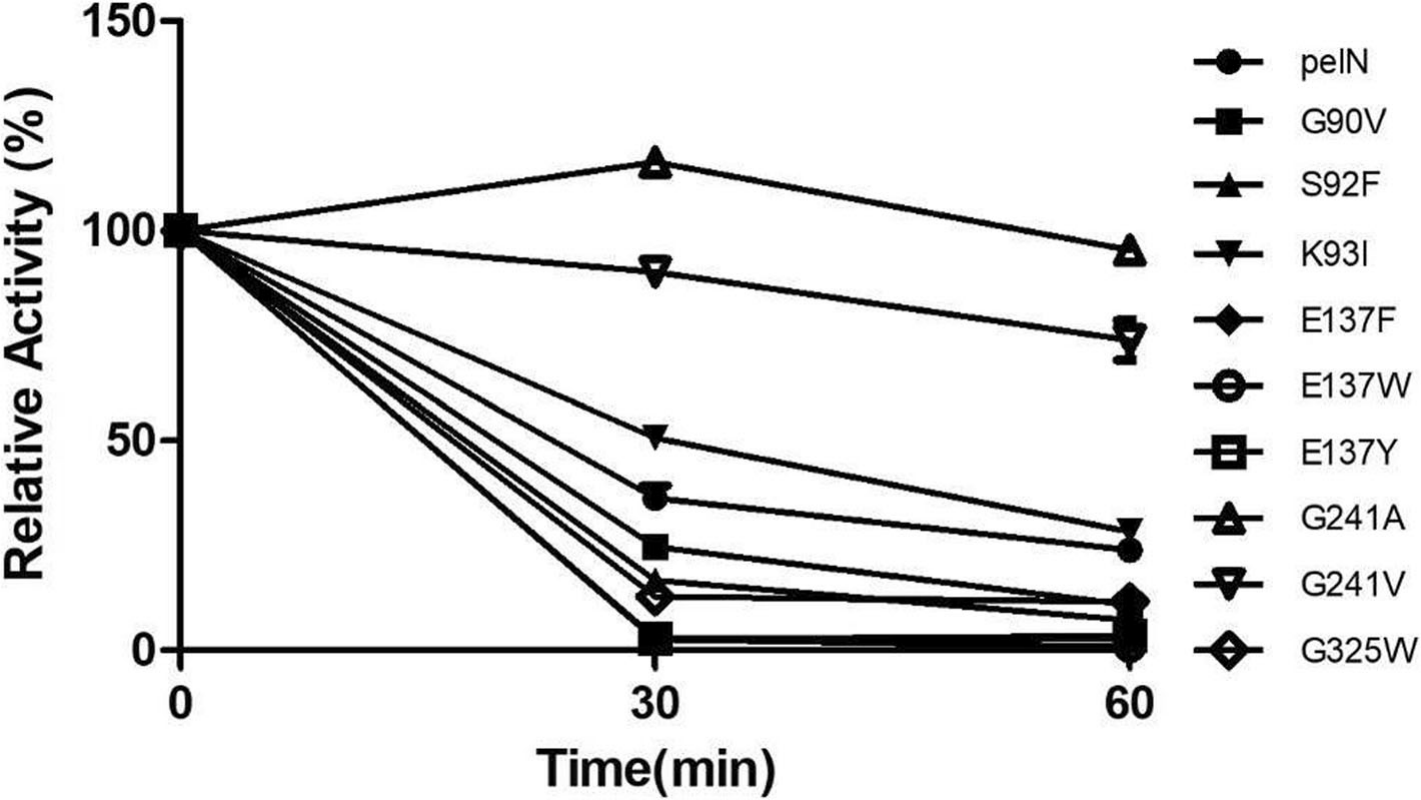
The residue enzymatic activities of wild PelN and PelN variants for incubation at 60 °C. The relative activity was determined with the same protein concentration. The initial enzymatic activity of all enzymes was defined as 100%. The residue enzymatic activities were determined after incubating for 30 min and 60 min. All experiments were repeated three times, and the mean values were expressed as ± SD

The residual enzymatic activities of the wild type and variants at 30 min and 60 min are shown in Fig. 2. The variants G241A and G241V presented higher enzymatic activity than the wild PelN. And the variants K93I exhibited almost same activity compared to the wild PelN. While others showed decreased enzymatic activity, meaning thermos-unstable than wild PelN. Therefore, these three variants were selected as candidates for the next study.

Three variants were overexpressed and purified using a Ni^2+^-chelating affinity column. SDS-PAGE analysis verified that no discernable difference existed between the molecular masses (~ 48 kDa) of wild PelN and variants (see Fig. S3). Then the protein characteristics were determined using purified proteins.

**Fig. 3:**
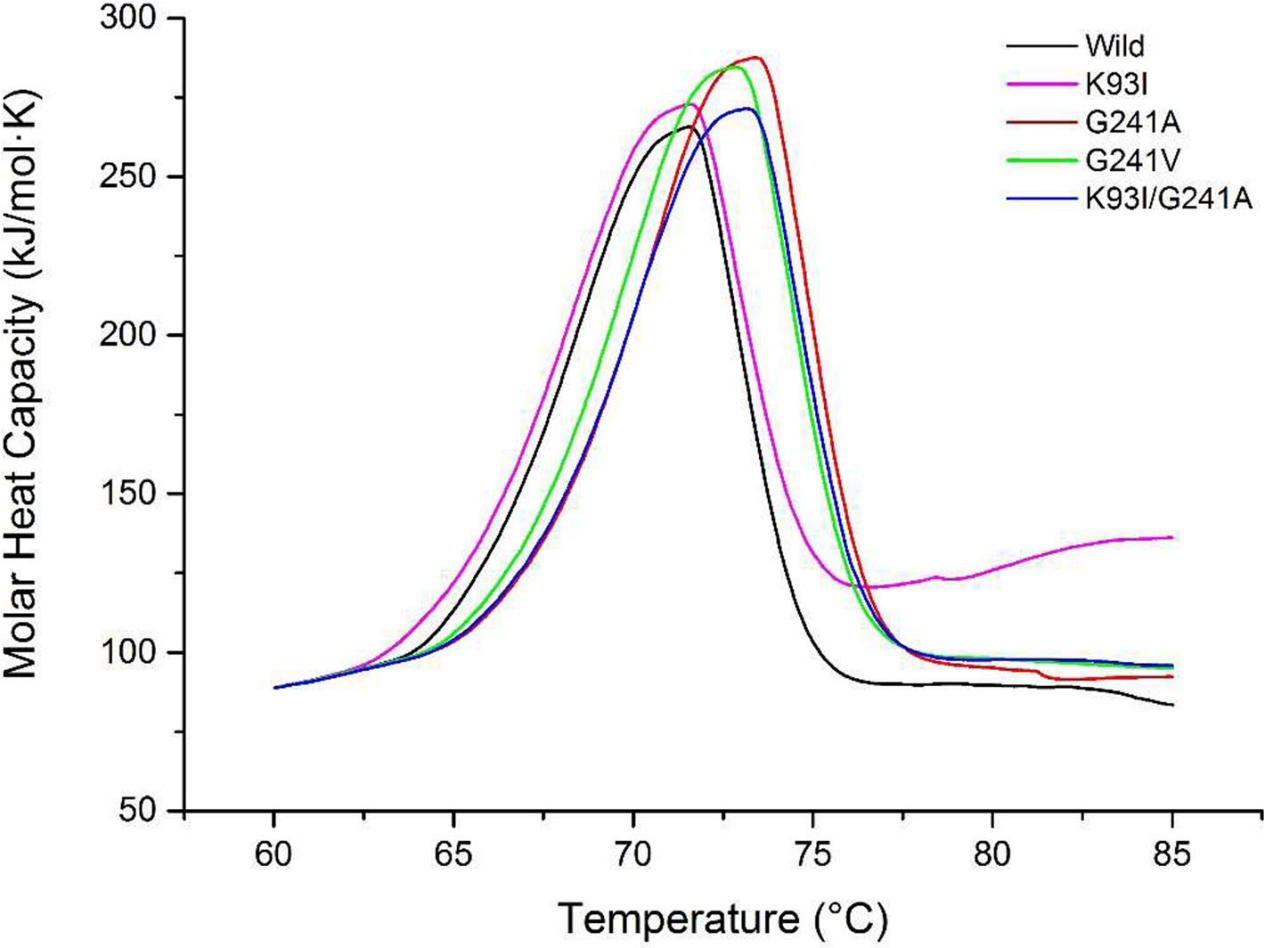
Thermostability of wild PelN and PelN variants determined by Nano DSC

The half-life of wild PelN was approximately 33.5 min at 60 °C (Table 1). The mutated enzymes G241A and G241V displayed longer half-lives by increasing 28.4 min and 13.6 min, respectively. The K93I mutation did not show any stability enhancement from the behavior of half-life at 60 °C. A previous study reported that the optimal temperature of K93I declined from 67.5 °C to 60 °C [11]. And same results were also shown in Fig. 3. Then, the mutations K93I and G241A were introduced simultaneously through site-directed mutagenesis to achieve a double-mutated variant (see Fig. S3). The half-life time of the double-mutated enzyme increased up to approximately 49.3 min, 15.9 min longer than that of the wild enzyme at 60 °C (Table 1), a remarkable improvement in thermostability.

**Table 1:** Summary of the thermostability characterization: specific activity, *t*_1/2_ (60 °C) and optimum temperature

Enzyme	Specific activity (U/mg)	*t*_1/2_ (60 °C) (min)	Optimum temperature (°C)
Wild type	1602 ± 124	33.47 ± 2.02	67.5
G241A	1449 ± 94	61.86 ± 3.94	67.5
G241V	1704 ± 119	47.09 ± 3.49	67.5
K93I/G241A	2707 ± 64	49.33 ± 0.14	60

A DSC assay was applied to verify the thermal effect on the protein conformational stability. The *T*_m_ values of mutant G241A, G241V and K93I/G241A increased by 1.8 ± 0.1 °C, 1.3 ± 0.1 °C and 1.6 ± 0.1 °C, respectively (Fig. 3), compared with the wild type PelN. This revealed that the replacement of glycine with alanine and valine significantly improved the thermostability of PelN.

**Fig. 4:**
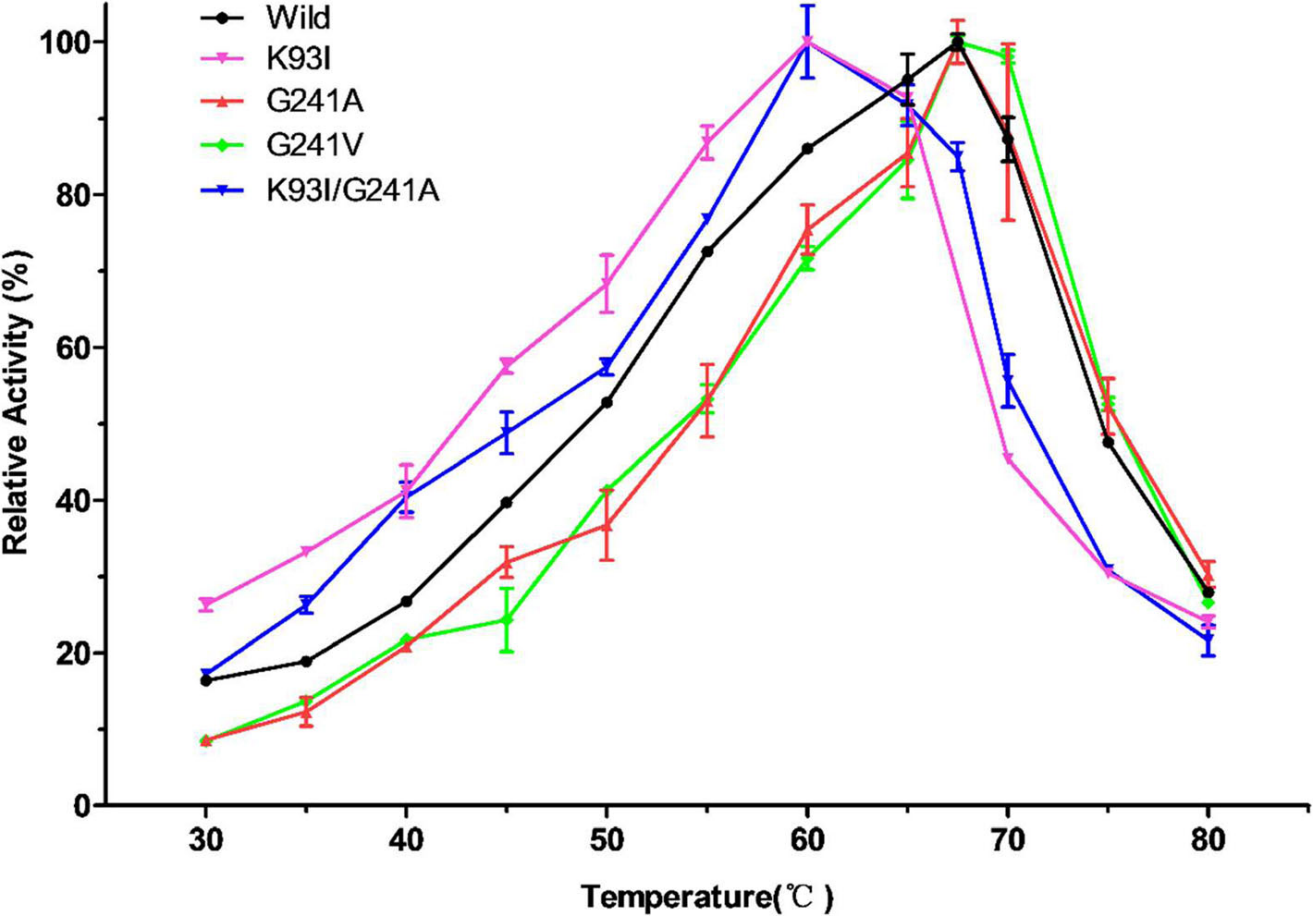
Temperature characteristics of wild PelN and PelN variants. The temperature with maximal enzymatic activity of K93I PelN variants decreased to 60 °C

Additionally, G241A and G241V have the same optimal temperature for enzymatic activity as wild PelN, while the optimal temperature of the double-mutated K93I/G241A followed that of K93I mutant and was determined to be 60 °C, declining from 67.5 °C of wild-type ones (Fig. 4). This temperature shift was a crucial advantage for industrial application. Heat is energy consumed in bioresource industries such as pectic wastewater treatment, textiles, and paper-making. Decreasing the treatment temperature means cost savings.

### Enzymatic characterization of the thermostable variants

We also detected the specific activities and catalytic parameters of mutants and wild-type PelN at 60 °C, as shown in the Table 1. The specific activity of K93I and double-mutated K93I/G241A significantly increased to 1.75-fold and 1.3-fold, respectively. The G241A and G241V mutants remained the same as the wild PelN. The Michaelis constants (*K*_m_ values) of all enzymes to the PGA substrate are given in Table 2. The *K*_m_ value of K93I mutant and double mutant K93I/G241A became lower than that of wild one, which indicated higher substrate-binding ability. Although the *K*_m_ values of G241A and G241V did not change, the catalytic constant (*k*_cat_) of G241V increased compared with that of PelN. As a result, G241A and G241V displayed higher catalytic efficiencies (indicated by *k*_cat_/*K*_m_) than PelN.

**Table 2:** Kinetic parameters of wild-type and mutated pelN

Enzyme	*K*_m_^a^ (g/l)	*k*_cat_^b^ (10^6^ s^−1^)	*k*_cat_/*K*m (10^5^ l/g·s)
Wild type	3.48 ± 0.35	3.29	9.45
K93I	0.74 ± 0.09	0.37	5.05
G241A	3.08 ± 1.25	3.54	11.50
G241V	3.98 ± 2.34	5.45	13.71
K93I/G241A	1.48 ± 0.04	0.78	5.26

^a^ *K*m, substrate dissociation constant

^b^ *k*_cat_, μmol unsaturated product equivalents per second per μmol protein

### Ramie degumming efficiency of variants

**Fig. 5:**
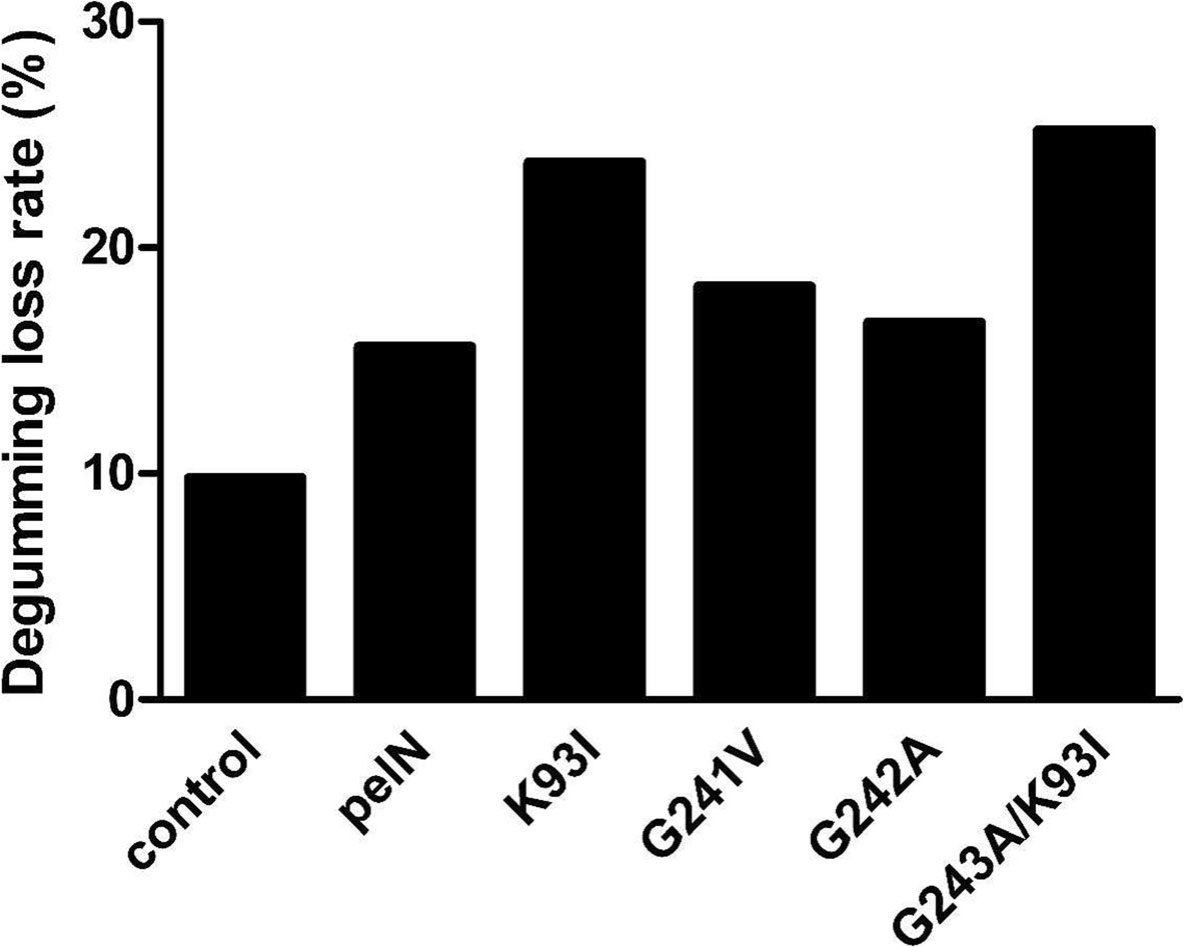
Ramie degumming efficiency of wild PelN and PelN variants

The application of PelN and variants in ramie degumming was also evaluated. The ramie was pretreated by NaOH and then degumming treatment with enzymes was processed at 50 °C for 12 h. All the tested variants have higher ramie degumming efficiency than wild PelN. As expected, when the ramie degumming was processed at relatively low temperature of 50 °C, the variant of K93I and double mutants G241A/K93I with lower optimal temperature exhibited excellent degumming ability (Fig. 5). These results indicated the possible industrial application of these pectate lyases.

### Double mutants is the best candidate for industrial application

Combined with the above results, the double mutants showed the advantage for industrial application in bioscouring. It showed three advantages than the wild PelN. Firstly, the double mutants showed longer half-life time of 15.9 min than the wild PelN. And DSC assay showed that the *T*_m_ of double-mutated enzyme also increased 1.6 °C versus that of the wild one (Fig. 3). Secondly, the specific activity of the double mutant increased 30% (Table 1). As to kinetic assay, the double mutant exhibited a lower *K*_m_ for the substrate PGA. Therefore, the double-mutated variant own higher substrate affinity (Table 2). Last, the optimal enzymatic temperature of the double mutant declined from 67.5 °C to 60 °C which was more suitable for the ramie degumming process.

## Discussion

Bioscouring is usually treated by a combination of chemical and biological methods because the fiber is softer and smoother than only using chemical treatment. Chemical method includes treatments with hot alkaline solutions before biological enzymatic treatment. Thermostable pectate lyase can treat more ramie by prolonging the enzymatic time and reducing cost in the ramie degumming process. A pectate lyase PelN was reported previously [1]. Compared to other reported pectate lyase, PelN exhibited an excellent characteristic at thermo-alkali stability [26–28]. PelN was also tested to put into biosouring application in industrial scale.

To better meet the requirement industrial application, researchers tried to improve the enzymatic characteristic of the pectate lyases. Wang et al. have doubled the half-life time at 50 °C of Pel168 from *Bacillus subtilis* [29]. Another Pel from *Bacillus pumilus* got longer half-life time to 13 h at 50 °C and higher optimal temperature to 75 °C after a single site mutation [10]. In this study, we constructed a series of proposed thermostable mutants by rational design. Finally, we obtained PelN-G241A and PelN-G241V with improved thermostability but without losing any enzymatic activity. Their half-life time increased by 28.36 and 13.62 min, respectively.

Ramie degumming is often treated by a mixture of several enzymes, including pectate lyase, Mannanase and Xylanase etc. To maximize the activity of the combined enzyme mixture, it is often to set the temperature of enzymatic degumming at 55–60 °C. Wild type PelN and its thermostable mutants have an optimal enzymatic temperature of 65 °C, with the result that they could not display the best enzymatic ability. We obtained a pectate lyase mutant PelN -K93I with a shifted optimal enzymatic temperature of 60 °C and improved specific activity to substrate PGA based on structural analysis. Generally, it is difficult to improve the thermostability and specific activity simultaneously [10, 30]. However, we fortunately obtained the PelN-K93I/G241A and PelN-K93I/G241V mutants that have an optimal enzymatic temperature of 60 °C and improved thermostability when combining PelN-K93I mutate with the thermostable mutants.

**Fig. 6:**
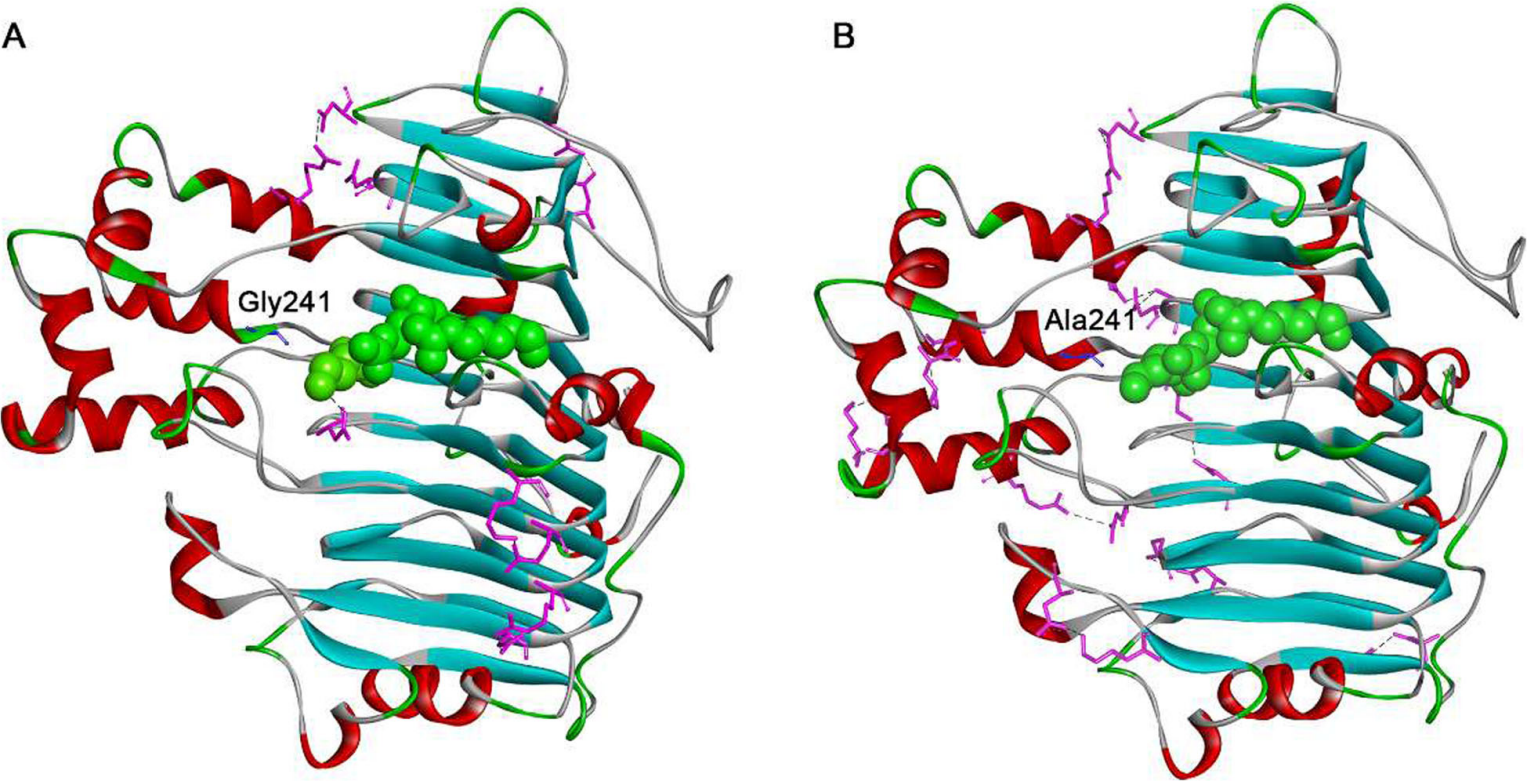
Comparisons of molecular interactions in PelNs in the process of G241A mutation. The molecular interactions were modeled by the Discovery Studio 4.1 program. The residues in which the molecular interaction has changed are colored pink. The residues are represented in the stick scheme. The hydrogen-bonding interactions are displayed by black dashed lines. **a** Wild-type, **b** G241A variant

**Fig. 7:**
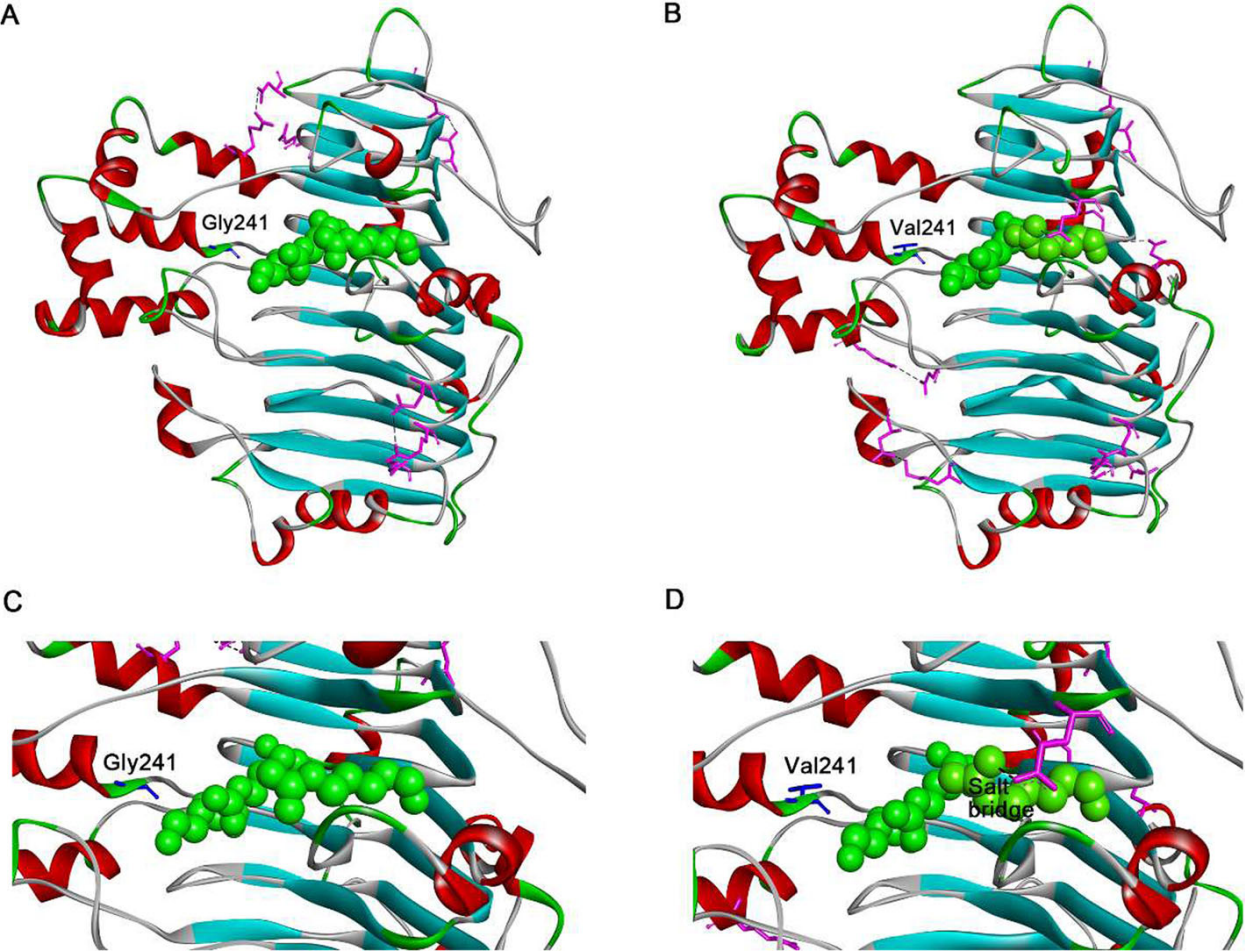
Comparisons of molecular interactions in PelNs in the process of mutation of G241V. The molecular interactions were modeled by the Discovery Studio 4.1 program. The residues in which the molecular interaction has changed are colored pink. The residues are represented in the stick scheme. The hydrogen-bonding interactions are displayed by black dashed lines. **a** Hydrogen-bonding interactions of the wild PelN, **b** hydrogen-bonding interactions of the G241V variant, **c** salt bridge interaction of the wild PelN, **d** salt bridge interaction of the G241V variant

Within the screened subset of proteins, we determined how the selected residue change affected overall protein stability by structure simulations. The 3D conformational models of PelN (Figs. 6 and 7 a, a), G241A (Fig. 6 b) and G241V (Fig. 7 b) were constructed using the SWISS-model online tool. Multiple protein sequence alignment indicated that the residues in the catalytic site included K245, R276 and R281 [31]. Gly 241 is in the close position to the catalytic residues. At position 241, a Gly to Ala substitution generated more salt bridges, increasing hydrophobic interactions (Fig. 6). There were 19 salt bridges in mutation G241A, more than in the wild PelN. The increased salt bridges were located on the protein surface (Fig. 6 b). A previous study emphasized the importance of amino acids and interactions on the surface for the stability [32, 33], because mobile loops on the protein surface act as initiation sites of protein unfolding. Solvent penetrates at these sites to the core and leads to protein unfolding [34]. In G241A mutants, new salt bridges located on the surface could not only make the flexible helixes and loops more rigid, but also increase the rigidity of the overall structure to improve the conformational stability. As a result, protein thermostability increased obviously. The details of the new salt bridges are shown in Table S4. The number of hydrophobic interactions in G241A was also calculated by Discovery Studio, which increased to 214 the wild PelN. This result indicated that 60% of protein stability was contributed by hydrophobic interactions [35]. The increased hydrophobic interactions also contributed to the thermostability of the G241A mutant. Therefore, both the protein stability [35] and thermostability of the G241A mutant were improved by more hydrophobic interactions.

The intramolecular interactions in G241V were accompanied by several new salt bridges (Fig. 7). From the PelN structure, Lys32 was on the β1 sheet and Gly241 was on a loop near the β1 sheet. The G241V mutation resulted in a new salt bridge between these two residues within 3.278 Å. The new salt bridge made the flexible loop near the β1 sheet more rigid. Another new salt bridge within 3.847 Å, cross-linked Asp6 on the α5 helix and Arg422 on the α12 helix, whose sites had a long compartment in sequence but were very adjacent in conformational space. The third new salt bridge within 2.726 Å, located on the protein surface between Lys76 and Asp79, might also contribute to the stability on high temperature. Notably, the new salt bridge within 3.855 Å formed between Glu134 and Arg206, compacted the structure via cross-linking the β8 sheet and α5 helix, and then improved the protein thermostability. Additionally, one new salt bridge (Arg281-Glu330) was around the catalytic residue Arg281 as shown in Fig. 7 c and d. This ordering effect caused by residue substitution tended to increase the rigidity of the loop and resulted in improved protein stability. In addition to new salt bridges, the increased number of hydrophobic interactions from 212 to 215 also contributed to the stability of PelN variants. These analyses demonstrated that the Gly to Val substitution at position 241 might be beneficial from both the formation of numbers of salt bridges and the increased hydrophobic interactions.

**Fig. 8:**
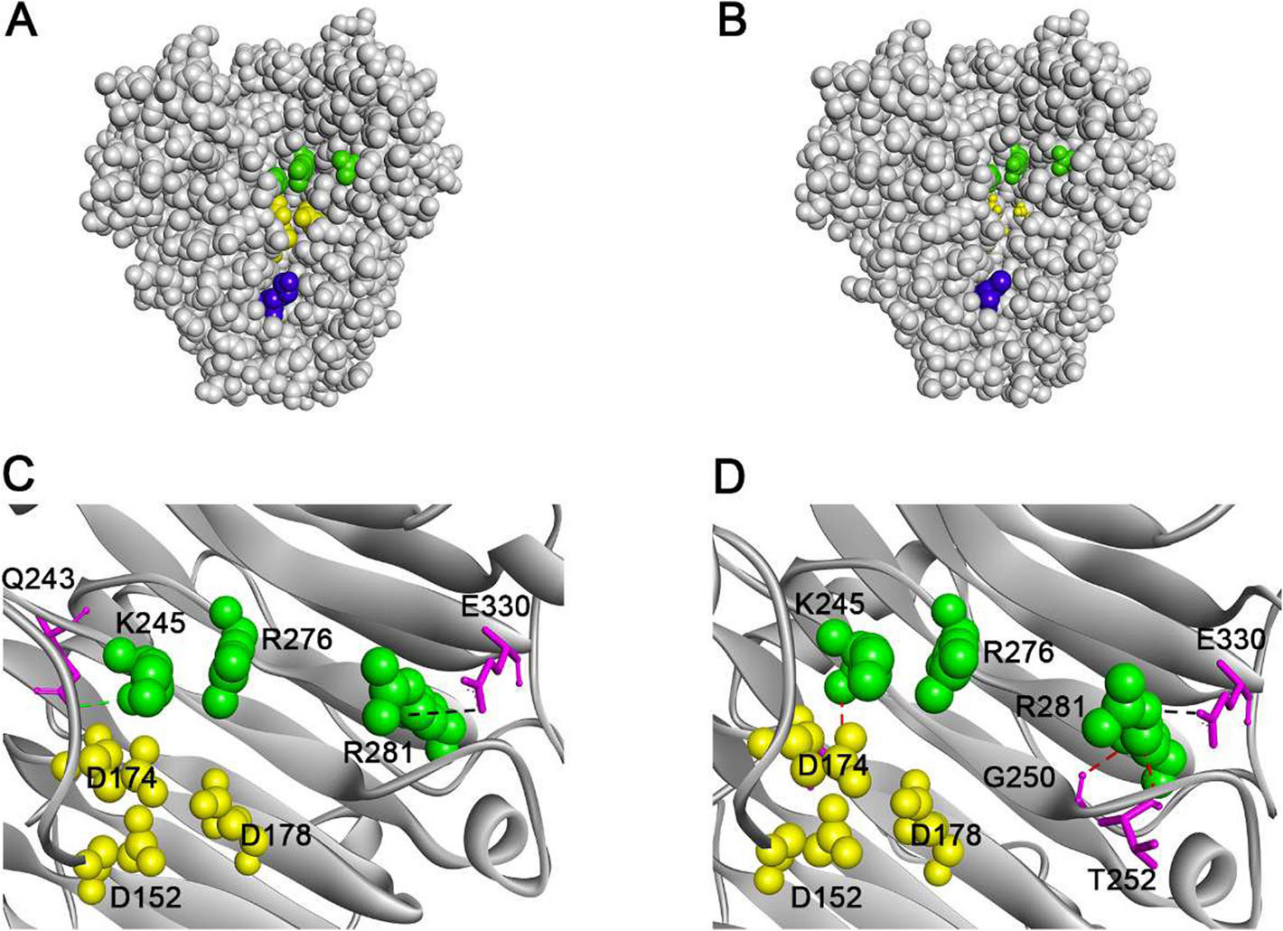
The molecular interactions have changed around Ca^2+^ in the active site that are caused by the K93I mutation. The models are displayed using the Pymol program. The residues in which the molecular interaction has changed are colored pink. The residues are represented in the stick scheme. The hydrogen-bonding interactions are displayed by black dash lines. **a** View of all residues in the wild PelN, **b** View of all residues in the K93I variant, **c** View of residue interactions around the Ca^2+^ activity site of the wild-type PelN, **d** View of residue interactions around the Ca^2+^ activity site of the K93I variant

The K93I mutants improved the specific activity [11] to substrate PGA by decreasing the maximal enzymatic temperature from 67.5 to 60 °C simultaneously. The mutation caused a change from basic to hydrophobic residues. Sequence alignment and structural superposition with BsPel (PDB ID: 3KRG) indicated that the plausible Ca^2+^-bind triad was D152, D174 and D178 [36]. Residue Lys93 was located close to the Ca^2+^-binding triad in the space structure (Fig. 8 a and Fig. 8 b). Nonbond interactions between catalytic and Ca^2+^-binding amino acids changed after hydrophobic Ile was introduced, as shown in Fig. 8 c and Fig. 8d. Some studies have proposed that conformational flexibility, especially around catalytic sites, tends to bring about an enzyme with high catalytic efficiency [37]. The substitution of Lys by Ile may cause a slight allosteric effect on the structure because the attractive charge within 5.425 Å between Arg281 and Glu330 disappeared while a new conventional hydrogen bond and two new carbon hydrogen bonds around Arg281 formed. On the other hand, the stronger rigidity between the catalytic residues in K93I might weaken the catalytic efficiency. Previous studies indicated that first calcium bound three aspartates (D152, D174, and D178), and D174 played a major role in the process of second calcium binding. This binding site included residues bound tightly to the substrate [36]. In K93I mutant, the conventional hydrogen bond within 3.319 Å between Lys245 and Gln243 disappeared and one carbon hydrogen bond within 3.785 Å between Lys245 and Asp175 formed. Thus, the newly-formed carbon hydrogen bond might affect the substrate affinity and optimal temperature because residue D175 coordinates the position of the Ca^2+^-binding site residues D174 and D178. The combined mutant K93I/G241A displayed the advantages of both the K93I mutant and the G241A mutant. Additionally, the ramie degumming efficiency of this variant indicated potential industrial application (Fig. 5).

## Conclusions

In conclusion, the alkaline PelN from the *Paenibacillus* sp. 0602 was successfully engineered by the PoPMuSiC algorithm and conformational analysis for increasing the protein thermostability in this study. The half-life of the selected mutant (G241A) increased 28.39 min longer than that of the wild type enzyme at 60 °C. The catalytic activity of another selected mutant (K93I) increased 74.59%. A new variant combining both K93I and G241A had a longer half-life time of 15.86 min at 60 °C and increased specific activity by 68.98%. Thus, the variant K93I/G241A should be more efficient in industrial applications with a higher specific activity at moderate temperature that could significantly reduce energy consumption. The results shed much light on structure-based proteomic rational design based on 3D conformational structure. Therefore, the combination of the PoPMuSiC algorithm, conformational analysis and mutation energy analysis with Discovery Studio would be practiced in the engineering of most industrial enzymes.

## Methods

### Material preparations

The alkaline pectate lyase gene *pelN* (GenBank accession no. KC351190) from *Paenibacillus* sp.0602 was obtained from the plasmid pET-22b-*pelN* in a previous study [1]. The PCR fragment of *pelN* was amplified and cloned into the plasmid pET-29a for expression, and the recombinant plasmid pET-29a-*pelN* was used for site-directed mutagenesis. The oligonucleotides for mutation are listed in Table 3. All the plasmids proliferated in *E. coli* DH5α as the host, and all the PLs were expressed in *E. coli* BL21 (DE3) as the host.

**Table 3:** List of primers

Name	Sequence	Description
PelN_F	GGAATTCCATATGGCGGGCAATGCAG	Nde1, PelN 5′
PelN_R	CCCTCGAGATAGCTCGTCTTC	Xho1, PelN 3′
G90V_F	GTGTTGAAGCAGACT**GTA**GTCAGCAAAA	G90V
G90V_R	CGTAATTTTGCTGAC**TAC**AGTCTGCTTC	G90V
S92F_F	AAGCAGACTGGTGTC**TTC**AAAATTACGG	S92F
S92F_R	GTCCACCGTAATTTT**GAA**GACACCAGTC	S92F
E137Y_F	TTCGATGAGCTGTGG**TAC**TGGGATGAGT	E137Y
E137Y_R	GGTGGACTCATCCCA**GTA**CCACAGCTCA	E137Y
E137F_F	TTCGATGAGCTGTGG**TTC**TGGGATGAGT	E137F
E137F_R	GGTGGACTCATCCCA**GAA**CCACAGCTCA	E137F
E137W_F	TTCGATGAGCTGTGG**TGG**TGGGATGAGT	E137W
E137W_R	GGTGGACTCATCCCA**CCA**CCACAGCTCA	E137W
D178F_F	TATGACGGACTTGTG**TTC**TCGAAAAAAG	D178F
D178F_R	GGTTCCTTTTTTCGA**GAA**CACAAGTCCG	D178F
D178Y_F	TATGACGGACTTGTG**TAC**TCGAAAAAAG	D178Y
D178Y_R	GGTTCCTTTTTTCGA**GTA**CACAAGTCCG	D178Y
D178C_F	TATGACGGACTTGTG**TGC**TCGAAAAAAG	D178C
D178C_R	GGTTCCTTTTTTCGA**GCA**CACAAGTCCG	D178C
D178V_F	TATGACGGACTTGTG**GTA**TCGAAAAAAG	D178V
D178V_R	GGTTCCTTTTTTCGA**TAC**CACAAGTCCG	D178V
G241A_F	ATTATTGCCATCTCT**GCG**TCGCAGAAAA	G241A
G241A_R	ACCTTTTTTCTGCGA**CGC**AGAGATGGCA	G241A
G241V_F	ATTATTGCCATCTCT**GTA**TCGCAGAAAA	G241V
G241V_R	ACCTTTTTTCTGCGA**TAC**AGAGATGGCA	G241V
G246W_F	GGTTCGCAGAAAAAA**TGG**CATCTTGTCG	G246W
G246W_R	CGCACCGACAAGATG**CCA**TTTTTTCTGC	G246W
G325W_F	GGGATTACCAGCAAC**TGG**GCTATTTCCA	G325W
G325W_R	TTCAGTGGAAATAGCCCAGTTGCTGGTA	G325W

Restriction sites are underlined and mutated nucleotides are in bold

Restriction endonucleases and DNA preparation kits in PCR were purchased from Transgen (Beijing, China). Protein quantification kit was the product of Bio-rad (Bio-Rad, USA). The Ni^2+^ Chelating column (HisTrap FF, GE Health, Uppsala, Sweden) was used in protein purification.

### Structure modeling of the PelN variants

The predicted structure of PelN variants were constructed on the online Swiss-Model server (https://swissmodel.expasy.org/) based on the known structure of wild PelN (5GT5). The final model showed good geometry [38]. The free energy (ΔΔG) change in the process of protein folding after amino acid replacement was predicted by the PoPMuSiC algorithm (https://dezyme.com/) [22]. The prediction showed that seven amino acid replacements (Glu137Tyr, Glu137Phe, Glu137Trp, Gly246Trp, Gly325Trp, Ser92Phe, Asp178Phe) had ΔΔG values of − 1.96, − 1.76, − 1.67, − 1.47, − 1.38, − 1.36, − 1.34, which were considered beneficial for protein stabilization. The software Discovery Studio 4.1 was also applied for the calculation of intramolecular interactions (salt bridges, hydrogen, and cation-pi interactions) in wild PelN and its variants.

### Protein preparation

The proteins in the study were prepared by strategies reported elsewhere [11]. First, the plasmids for expressing proteins were constructed by the PCR-based site-directed mutagenesis method [25], which was carried out with a 2 × EasyTaq PCR SuperMix (+dye) kit following the suggested protocol. PCR was performed using the plasmid pET-29a-*pelN* as the template DNA. The plasmids were confirmed through DNA sequencing. Then, confirmed plasmids were expressed in *E. coli* BL21 (DE3) to obtain proteins. The proteins with His-tags at the C-terminus were purified by Ni-NTA chelating chromatography and quantified with the Bradford method [12] using bovine serum albumin as the standard. The degree of purity was assessed by SDS-PAGE on a 12% gel.

### Enzymatic activity determination

Alkaline pectate lyase activity was measured as the release of unsaturated oligogalacturonates during cleavage of PGA and measured as reported [39]. The substrate solution was prepared by dissolving 0.2 g PGA in 100 mL glycine-NaOH buffer (pH 9.8, 50 mM) with additional 0.5 mM CaCl_2_. Enzyme solutions were added to 2 mL substrate solution and incubated at 45 °C for 15 min. Absorbance of the mixture at 235 nm was determined after the reaction was terminated by adding 3 mL of 0.03 M H_3_PO_4_. One unit of enzymatic activity was defined as the amount of enzyme required for the release of 1 μmol of unsaturated product per minute under the assay conditions.

### Selection of thermostable variants

The supersonic supernatants of *E. coli* expressing wild PelN or mutants were diluted with lysis buffer and assayed for enzymatic activity. The crude enzyme solutions were heat treated at 60 °C for the following time intervals: 0, 30, and 60 min. The enzymes in each time interval were quickly transferred and placed on ice for 10 min, and the residual enzyme activities were assayed. Compared with the wild PelN, variants maintained a remarkable increase in activity in residual enzyme and were considered candidate mutants that can enhance thermostability.

### Protein characteristic

Enzymatic characteristics such as *K*_m_, *k*_cat_, and *k*_cat_/*K*_m_ of the wild PelN and variants were determined by an enzymatic analysis scheme with different PGA concentrations. The reaction time was set for 1 min to try to measure the initial reaction rates. All quadruplicate measurements data were analyzed by GraphPad Prism software (version 5.0 for Windows, USA), and the kinetic parameters were calculated based on a nonlinear regression analysis. The optimal temperature and pH for enzyme activity were determined by the standard enzymatic analysis method. The assaying buffer used both Tris-HCl (pH 8.0–9.0, 50 mM) and glycine-NaOH (pH 9.0–11.0, 50 mM) in optimal pH determination.

### Thermostability measurement

The irreversible thermal incubation of the purified enzyme was measured by incubation in 20 mM phosphate buffer (pH 7.6) for setting intervals at 60 °C. The enzymatic thermostability was measured by the biological parameter half-life (*t*_1/2_) [40]_._

Melting temperature (*T*_m_) is another marker of proteomic thermostability. The *Tm* value was identified by a Nano differential scanning calorimetry (DSC) (Model 5100, Calorimetry Science Corporation, Utah, USA) with a scan rate of 1 °C/min at 3 atm. The purified proteins with a concentration of 0.25 mg/ml in 20 mM phosphate buffer (pH 7.6) were analyzed with increasing temperature from 35 °C to 90 °C. All data processing was performed using affiliated software.

### Degumming experiment

The ramie degumming efficiency of the variants was determined using the following procedure. A 1 g ramie was treated with 30 units/mL purified PelN and variants in 50 mL glycine-NaOH buffer (pH 9.8) at 50 °C for 12 h. In the control experiment, no enzyme was added. The ramie was washed with water and dried at 115 °C after enzymatic treatment. The ramie degumming efficiency was evaluated by the weight loss of ramie fibers.

## Supplementary Information

**Additional file 1: Table S1**. Stability prediction of mutation by Discovery Studio 4.1. **Table S2**. Calculation of △△G by PoPMuSiC. **Table S3**. Calculation of mutation energy by Discovery Studio 4.1. **Table S4**. Data about the 3D conformational models of pelN, and its mutants modeled by SWISS-MIDEL tool. **Table S5**. New-generated salt bridges in mutation G241A. **Figure S1.** SDS-PAGE analysis of the pelN and variants expression. **Figure S2.** The enzymatic activity of the pelN and variants cultures. **Figure S3.** SDS-PAGE analysis of the purified pelN and variants. Lanes: Marker, molecular standard; 1, lysate of cells; 2, supernatants; 3, purified proteins.

## Data Availability

The datasets used and/or analyzed during the current study are available from the corresponding author upon reasonable request.
