# Supplementary material for: Rational design and structure-based engineering of alkaline pectate lyase from Paenibacillus sp. 0602 to improve thermostability
Source: BMC Biotechnol. 2021 May 3;21:32. doi: 10.1186/s12896-021-00693-8 (PMC8091735; doi:10.1186/s12896-021-00693-8)
Supplement: Supplementary file 1 — Additional file 1: Table S1. Stability prediction of mutation by Discovery Studio 4.1. Table S2. Calculation of △△G by PoPMuSiC. Table S3. Calculation of mutation energy by Discovery Studio 4.1. Table S4. Data about the 3D conformational models of pelN, and its mutants modeled by SWISS-MIDEL tool. Table S5. New-generated salt bridges in mutation G241A. Figure S1. SDS-PAGE analysis of the pelN and variants expression. Figure S2. The enzymatic activity of the pelN and variants cultures. Figure S3. SDS-PAGE analysis of the purified pelN and variants. Lanes: Marker, molecular standard; 1, lysate of cells; 2, supernatants; 3, purified proteins. [file 12896_2021_693_MOESM1_ESM.docx]

**Supplementary Materials for submission to Applied Microbiology and Biotechnology**

**Rational design and structure-based engineering of alkaline pectate lyase from *Paenibacillus* sp. 0602 to improved thermostability**

Zhanping Zhou^a^, Xiao Wang^b^*

^a^Tianjin Sinonocy Biological Technology Co. Ltd., Tianjin, 300308, China

^b^Nanfang College of Sun Yat-Sen University, Guangdong 510970, China

* **Correspondence Author:** Xiao Wang

Address: Email: wangx@nfu.edu.cn

Nanfang College of Sun Yat-Sen University, Guangdong 510970, China

Email addresses: [wangx@nfu.edu.cn](mailto:wangx@nfu.edu.cn)

Tel.: +86-020-61787104; Fax: +86-020-61787104

Table S1 Stability prediction of mutation by Discovery Studio 4.1

| Index | Mutation | Mutation Energy (kcal/mol) | Effect |
| --- | --- | --- | --- |
| 1 | Gly90→Val | -1.77 | Stabilizing |
| 2 | Gly241→Ala | -1.14 | Stabilizing |
| 3 | Gly241→Val | -0.91 | Stabilizing |
| 4 | Pro219→Val | -0.27 | Neutral |
| 5 | Pro219→Ala | 0.78 | Destabilizing |
| 6 | Gly90→Ala | 1.11 | Destabilizing |
| 7 | Pro84→Val | 1.23 | Destabilizing |
| 8 | Pro84→Ala | 1.95 | Destabilizing |

**Table S2 Calculation of △△G by PoPMuSiC**

| Index | Mutation | △△G (kcal/mol) | Index | Mutation | △△G (kcal/mol) |
| --- | --- | --- | --- | --- | --- |
| 1 | E137Y | -1.96 | 16 | D178I | -1.2 |
| 2 | E137F | -1.76 | 17 | E53I | -1.19 |
| 3 | E137W | -1.67 | 18 | S328F | -1.17 |
| 4 | G246W | -1.47 | 19 | E137V | -1.14 |
| 5 | G325W | -1.38 | 20 | G325Y | -1.14 |
| 6 | S92F | -1.36 | 21 | S331G | -1.14 |
| 7 | D178F | -1.34 | 22 | S92V | -1.13 |
| 8 | S92W | -1.26 | 23 | E137M | -1.13 |
| 9 | S92I | -1.26 | 24 | S92C | -1.09 |
| 10 | D178Y | -1.25 | 25 | D178C | -1.09 |
| 11 | S92Y | -1.22 | 26 | D178V | -1.09 |
| 12 | D165I | -1.21 | 27 | E137H | -1.04 |
| 13 | D178W | -1.21 | 28 | K931 | -1.03 |
| 14 | E337F | -1.21 | 29 | E337Y | -1.03 |
| 15 | D165V | -1.2 | 30 | D165L | -1.02 |

Table S3 Calculation of mutation energy by Discovery Studio 4.1

| **Index** | **Mutation** | **Mutation Energy** (kcal/mol) | **Effect of Mutation** | **VDW Term** | **Electrostatic Term** | **Entropy Term** |
| --- | --- | --- | --- | --- | --- | --- |
| 1 | Lys93→Ile | -2.67 | Stabilizing | -1.89 | -1.45 | -1.25 |
| 2 | Asp178→Phe | -2.24 | Stabilizing | -1.13 | -3.36 | 0.01 |
| 3 | Asp178→Tyr | -1.77 | Stabilizing | -1.47 | -2.71 | 0.4 |
| 4 | Asp178→Cys | -1.74 | Stabilizing | 4.09 | -4.98 | -1.62 |
| 5 | Asp178→Val | -1.66 | Stabilizing | 1.97 | -4.74 | -0.34 |
| 6 | Asp165→Ile | -1.56 | Stabilizing | 1.85 | -4.89 | -0.06 |
| 7 | Gly246→Tyr | -1.51 | Stabilizing | -4.07 | 0.96 | 0.06 |
| 8 | Asp178→Ile | -1.36 | Stabilizing | 1.72 | -4.33 | -0.07 |
| 9 | Ser92→Trp | -0.68 | Stabilizing | -2.52 | 1.44 | -0.17 |
| 10 | Asp165→Leu | -0.56 | Stabilizing | 3.61 | -4.96 | 0.15 |

Table S4 data about the 3D conformational models of pelN, and its mutants modeled by SWISS-MIDEL tool

|  | K93I | G241A | G241V | K93I/G241A |
| --- | --- | --- | --- | --- |
| **Template** | 5gt5.1.A | 5gt5.1.A | 5gt5.1.A | 5gt5.1.A |
| **Seq Identity** | 99.55 | 99.55 | 99.55 | 99.33 |
| **Oligo-state** | homo-dimer | homo-dimer | homo-dimer | homo-dimer |
| **QSQE** | 0.55 | 0.56 | 0.56 | 0.55 |
| **Found by** | HHblits | HHblits | HHblits | HHblits |
| **Method** | X-ray | X-ray | X-ray | X-ray |
| **Resolution** | 1.45Å | 1.45Å | 1.45Å | 1.45Å |
| **Seq Similarity** | 0.61 | 0.61 | 0.61 | 0.61 |
| **Range** | 3 - 446 | 3 - 446 | 3 - 446 | 3 - 446 |
| **Coverage** | 1.00 | 1.00 | 1.00 | 1.00 |
| **Description** | Pectate lyase | Pectate lyase | Pectate lyase | Pectate lyase |

Table S5 New-generated salt bridges in mutation G241A

| Name | Distance | From | From Chemistry | To | To chemistry |
| --- | --- | --- | --- | --- | --- |
| LYS32 - GLU24 | 3.46983 | LYS32:NZ | H-Donor | GLU24:OE1 | H-Acceptor |
| LYS76 - ASP79 | 2.7011 | LYS76:NZ | H-Donor | ASP79:OD1 | H-Acceptor |
| LYS113 - ASP57 | 3.47741 | LYS113:NZ | H-Donor | ASP57:OD2 | H-Acceptor |
| LYS194 - GLU131 | 3.7875 | LYS194:NZ | H-Donor | GLU131:OE2 | H-Acceptor |
| ARG206 - GLU134 | 3.94525 | ARG206:NH2 | H-Donor | GLU134:OE2 | H-Acceptor |
| LYS215 - GLU212 | 3.15378 | LYS215:NZ | H-Donor | GLU212:OE2 | H-Acceptor |
| LYS233 - GLU234 | 3.69646 | LYS233:NZ | H-Donor | GLU234:OE2 | H-Acceptor |
| LYS271 - ASP272 | 3.32252 | LYS271:NZ | H-Donor | ASP272:OD2 | H-Acceptor |
| LYS271 - ASP296 | 2.81868 | LYS271:NZ | H-Donor | ASP296:OD2 | H-Acceptor |
| ARG298 - ASP374 | 3.52257 | ARG298:NH2 | H-Donor | ASP374:OD2 | H-Acceptor |


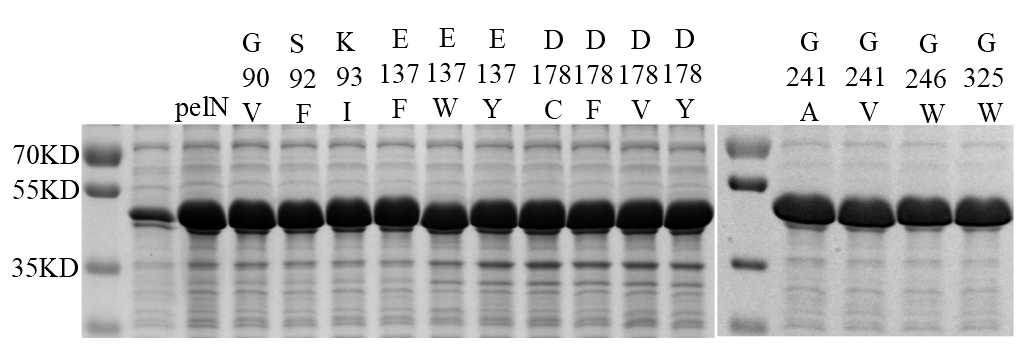


Fig. S1 SDS-PAGE analysis of the pelN and variants expression.


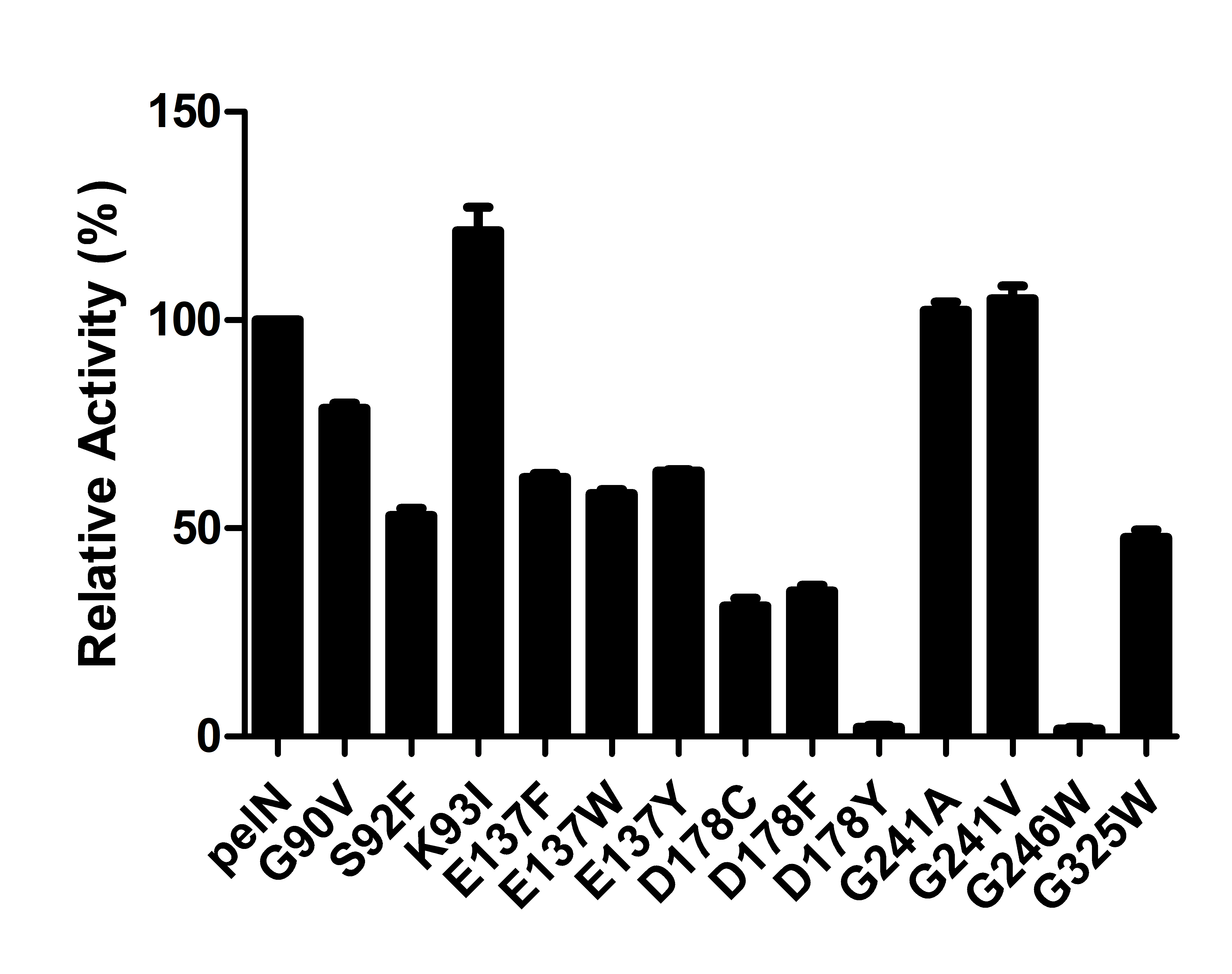


Fig. S2 The enzymatic activity of the pelN and variants cultures.


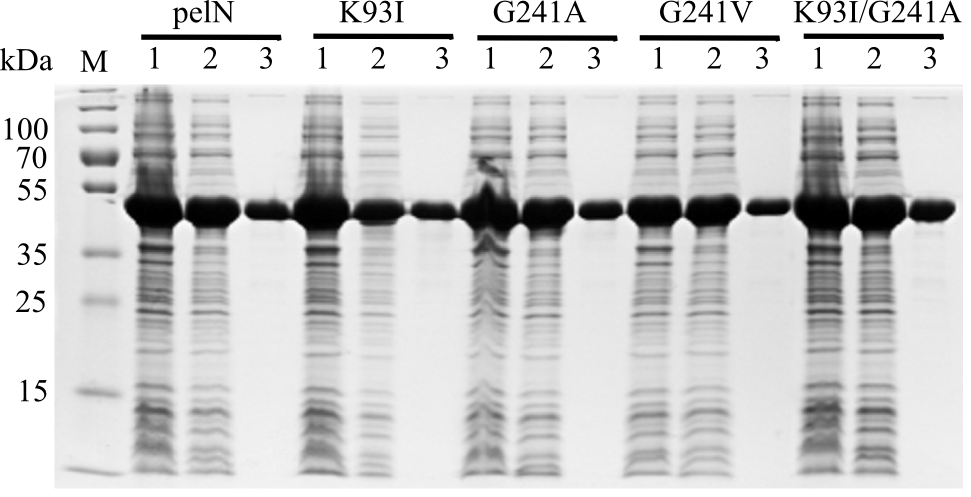


Fig. S3 SDS-PAGE analysis of the purified pelN and variants.

Lanes: Marker, molecular standard; 1, lysate of cells; 2, supernatants; 3, purified proteins.
